# Financial Abuse in a Banking Context: Why and How Financial Institutions can Respond

**DOI:** 10.1007/s10551-023-05460-7

**Published:** 2023-06-02

**Authors:** Ayesha Scott

**Affiliations:** Faculty of Business, Economics & Law, Auckland University of Technology (AUT), Auckland, New Zealand

**Keywords:** Economic abuse, Financial abuse, Intimate partner violence, Consumer vulnerability, Banking, Systemic harms

## Abstract

Intimate Partner Violence (IPV) is a global social problem that includes using coercive control strategies, including financial abuse, to manage and entrap an intimate partner. Financial abuse restricts or removes another person’s access to financial resources and their participation in financial decisions, forcing their financial dependence, or alternatively exploits their money and economic resources for the abuser’s gain. Banks have some stake in the prevention of and response to IPV, given their unique role in household finances and growing recognition an equitable society is one inclusive of consumers with vulnerabilities. Institutional practices may unwittingly enable abusive partners’ financial control as seemingly benign regulatory policy and tools of household money management exacerbate unequal power dynamics. To date, business ethicists have tended to take a broader view of banker professional responsibility, especially post-Global Financial Crisis. Little scholarship examines if, when and how a bank should respond to societal issues, such as IPV, traditionally outside their ‘remit’ of banking services. I extend existing understandings of ‘systemic harm’ to conceptualise the bank’s role in addressing economic harm in the context of IPV, viewing IPV and financial abuse through a consumer vulnerability lens to translate theory into practice. Two in-depth stories of financial abuse further illustrate the active role banks can and should take in combating financial abuse.

## Introduction

Violence against women is a global scourge, a complex social problem with wide-ranging and expensive harms for communities, economies, and society. Intimate partner violence (IPV), often referred to as family violence, domestic violence, or domestic abuse, is predominantly violence against women (World Health Organisation, 2010). It encompasses a range of coercive controlling behaviours, including physical, sexual, psychological and emotional, and economic and financial abuse, to manage and entrap an intimate partner (Tolmie et al., 2018). ‘Traditional’ forms of IPV, for example, physical abuse, are relatively well-researched if not easily solved, nor wholly understood, with economic and financial abuse long considered an unfortunate consequence of experiencing other forms of abuse. Now, economic and financial abuse is understood to be a standalone form of violence (Adams et al., 2008), with prevalence studies suggesting 1 in 5 women face this type of violence (Sharp-Jeffs, 2015, U.K. population-based study).

To date, governmental, policy, and scholarship have focused on integrating health, legal and justice, and social systems to address IPV and coercive control. Drawing the financial system and its institutions into these efforts, however, is in its infancy. Social service providers are pushing global momentum with, for example, Women’s Information and Referral Exchange (WIRE) in Australia and Surviving Economic Abuse (SEA) in the U.K. working toward banking solutions. Similarly, in Aotearoa New Zealand (N.Z.), the social sector (e.g., Shine, Women’s Refuge and Good Shepherd NZ) is aiding victims-survivors facing the realities of this oppressive form of violence and working with motivated banks.

Banks hold a unique and powerful position in an intimate relationship, power yet to be fully tapped into by the bank to both avoid harm and do good. Notable exceptions exist, both at an individual banking level (BNZ in N.Z., NAB, CBA and Westpac in Australia, and RBS NatWest and Lloyd’s Banking Group in the U.K.) and industry level, including the Australian Bankers’ Association (2016) Industry Guideline for financial abuse, and the U.K. Finance (2018) Financial Abuse Code of Practice. The Co-Operative Bank (U.K.) supported the economic abuse-focused prevalence study above and its update (Butt, 2020).

Business ethicists have tended to take a broader view of banker professional responsibility. The focus on banking stability and systemic importance is constrained for the most part to the “too big to fail” setting, especially post-Global Financial Crisis (GFC), see for example Moggia (2019), Linsley and Slack (2013), and EY (2014). Corporate social responsibility (CSR) is also not a new topic in ethics (see Scholtens, 2009 and Pérez & del Bosque, 2012 for banking examples), nor finance (McGuire et al., 1988). de Jonge (2018) places a feminist lens on CSR, exploring the responsibility of workplaces to support their employees experiencing violence. More generally, how employers may best support staff experiencing IPV is surveyed by MacGregor et al. (2020), suggesting there is no ‘one size fits all’ approach. However, scholarship is sparse on if, when and how a financial institution should respond to societal issues, such as IPV, traditionally outside their ‘remit’ of financial services.

I aim to fill this gap by bringing together two distinct but interrelated strands of the business ethics literature to argue *why* and illustrate *how* the financial institution, specifically the retail bank, plays a vital role in society’s response to financial abuse as IPV. The first is systemic harm (Armour & Gordon, 2014), that is, the financial institution as systemically important to society. Therefore, its actions are felt beyond its direct interest groups such as consumers and shareholders. By focussing on the bank’s relationship with a consumer, I expand Armour and Gordon’s (2014) systemic harms into the realm of retail banking from the post-GFC reform context mentioned above. The second concept used is consumer vulnerability, an idea of increasing prominence for all corporates but arguably even more significant for systemically important institutions.

Herzog (2019) cites Armour and Gordon (2014) for the term “systemic harms” and suggests two paths: ‘narrow’ and ‘broad’. Herzog addresses the so-called narrow path, opting to guide the prevention of additional harm to society. I address ‘broad’ duties *and* the narrow, encompassing both positive and negative avoidance of harm. In the former, providing space for a dedicated domestic and family violence response within the bank’s core business allows a targeted and hopefully more impactful approach. For the latter, an example may be protecting an existing customer’s safety by ensuring their mailing address cannot be accessed by their abuser. I propose the role of the bank is relevant both as remedy and prevention: providing remedy to a victim-survivor of IPV and, specifically, financial abuse within a framework of consumer vulnerability, *and* can play an active role in shaping a new path forward for all consumers’ healthy financial relationships.

Like CSR, consumer vulnerability has been explored in a variety of contexts. Industry bodies and regulators have created codes of practice and guidelines for various sectors, banking and financial services included, and scholars have a long tradition of highlighting vulnerabilities and their impacts. In one such study, Graham (2018) examines case studies of consumer vulnerability responses from the service provider's perspective (energy and banking). They examine policy frameworks, complaints procedures, and remedial powers given to the consumer-facing staff at the participant organisations; however, they do not explicitly address violence against women or IPV. Like Graham, the perspective presented here is that of the victim-survivor as an existing or prospective bank customer. While it is reasonable to expect a bank may match its outward stance with an internal support framework, the scope is on the consumer/bank relationship rather than the employee/employer context.

While this article focuses on the retail banking sector, related questions of consumer inclusion and exclusion (Akaah, 1992, Meyer, 2018 and Miller & Stovall, 2019) are highly relevant to all business sectors. An increasingly complex and fast-paced digital world widens the scope of a corporation’s influence beyond its direct network. I invite readers to define “financial institutions” in the broadest sense, with the argument made here transferrable to any consumer-facing organisation, especially those providing economic resources necessary to live a full life in contemporary society. Examples that immediately spring to mind are telecommunication and utility companies, as both industries also face questions of what consumer vulnerability means in an operational setting.

To the best of my knowledge, this is the first (academic) article to theorise the argument for retail banks taking an active role in combating economic harm in intimate partnerships. Further, framing victim-survivors of financial abuse and IPV as vulnerable provides a framework for putting theory into practice. I present two juxtaposing examples, one positive and one negative, to illustrate the real consequences of bank and financial institution inaction in this space. The stories are drawn from a collection of twenty-three women’s stories of violence gathered over two related qualitative studies, both under institutional ethics committee approval (AUT Ethics Committee AUTEC Reference Numbers 18/85 and 18/214). The article is conceptual rather than empirical and is not a complete retelling of their complex stories nor a presentation of the findings from those projects. Rather, the examples underscore the importance of considering victim-survivors as vulnerable and the financial institution as systemically important in preventing further harm and actively responding to financial abuse as part of a collective societal response.

## What is Financial Abuse?

Financial abuse is defined as behaviour that restricts, controls, exploits, or removes another person’s access to money, economic resources, or participation in financial decisions. While the literature tends to use the terms ‘economic abuse’ and ‘financial abuse’ interchangeably, recent work by Sharp-Jeffs (2021) clearly describes financial abuse as one facet of the more broadly defined economic abuse. The former focuses on monetary and financial resources, whereby the latter takes a wider view of economic resources, including housing, employment, and education. There are instances of overlap, such as credit ratings or mortgages, which may impact housing (an economic resource). Neither economic nor financial abuse require physical proximity to perpetrate (Stark, 2007), allowing abuse to continue unabated post-separation and severely restricting victims’ ability to move on with their lives (Scott, 2020a). Both are mechanisms of partner and systemic entrapment (Elizabeth, 2015; Jury et al., 2017; Tolmie et al., 2018; Walby & Towers, 2017). In their work on “nonviolent coercive control”, Crossman et al. (2016) find victim-survivors reported higher levels of fear post-separation than those in their “violent” and “non-violent” comparison groups. Additionally, online platforms (including banking apps) and social media also aid financial control and stalking behaviours, giving rise to newer methods of abuse such as so-called transaction abuse (Brook, 2020).

Consequences of financial abuse include trauma-related health issues, poverty, debt, lost income, and unemployment (O’Leary-Kelly et al., 2008), restricting a victim’s ability to end the relationship and seek safety for themselves and their family. Inequity in systems, including legal and justice (Elizabeth et al., 2012), social (Bennett & Sung, 2013), and financial (Sharp-Jeffs, 2015), exacerbate violence and contribute to wider ‘systemic entrapment’ as inequity intersects with disadvantage. Examples of disadvantage include gender, ethnicity, immigration, ongoing impacts of colonisation for Indigenous women, socioeconomic status, health, and financial capability—each is compounded by societal norms, traditional gender roles, and the taboo of open money conversations. Thus, any response to financial abuse, and IPV, is increasingly understood to require a collective shift from victim empowerment toward holistic, system-wide approaches involving the whole of society and its communities (Family Violence Death Review Committee, 2016).

At the time of writing, the global community is facing ongoing uncertainty due to the COVID-19 pandemic, with varying degrees of high financial stress, isolation and forced proximity. Financial distress and hardship have increased, leading to reports of increased conflict over money matters within households (Galicki, 2020). Preliminary studies show rates of family and intimate partner violence have increased, as movement restrictions heighten barriers to safety for victim-survivors locked down with their abusers (see, e.g., Franks, 2020, for N.Z.-based data; Stubbs-Richardson and Sinclair, Dec 5 2020 for U.S. context). Krigel and Benjamin (2020) provide (pre-COVID) insights into the transitional path from physical through to economic abuse, suggesting the typical experience of IPV is not only complex but changes form over the course of the relationship. As nations flatten their pandemic curves, ease lockdown restrictions (Whyte, 2020), emergency financial support ends (e.g., wage subsidies and mortgage holidays), and the economic impact of the pandemic becomes clear, it is logical to hypothesise other forms of IPV and coercive control (including financial abuse/control) may become more prevalent. Combined with general financial uncertainty and instability, a higher number of retail banking consumers are at risk of vulnerability and for victim-survivors of IPV, the risk is acute.

## Banker Responsibility: Consumer Vulnerability & Systemic Harms

### The Retail Bank as Systemically Important

Banks are corporate institutions of systemic importance. That is, it is in the interests of a nation and its citizens that their banking system functions well, as the impacts of failure are adverse consequences for everyone in that economy, including those outside that one bank’s direct business (Armour & Gordon, 2014; Herzog, 2019). In the context of banking institutions, stakeholders are not simply those with direct relationships, such as shareholders and customers, but rather the bank is, and should be, invested in the society in which they operate (de la Cuesta-González et al., 2020).

The ethics literature provides much discussion of banks concerning their role and responsibility as systemically important actors in a wider economic system, with post-GFC reforms a case in point (see, e.g., EY, 2014, Moggia, 2019, and Linsley & Slack, 2013). However, scholarship is limited on banking ethics and household or consumer issues outside the vulnerability context examined below, providing little guidance on if, when and how a financial institution should respond directly to social problems, such as IPV. For households, it is banks lending to mortgage holders to provide shelter, finance businesses and thus allow employment, and facilitate the day-to-day money management of individuals and their families. In extraordinary times, such as those of the COVID-19 pandemic, it is banks providing temporary relief for households through adjustments to debt repayment terms in the event an individual—through job loss,  income reduction or extended leave/furlough—is struggling to service their debt. It is important to conceptualise the bank or financial institution as systemically powerful not only for a society and its economy, but also as wielding unique power within a household or intimate partnership—power that can inadvertently cause harm or do good.

Money can indicate other aspects of an intimate relationship, especially the power dynamics between partners (Cantillon et al., 2016; Heimdal & Houseknecht, 2003; Pahl, 1989, 1995; Singh & Lindsay, 1996; Vogler & Pahl, 1994). An individualistic approach to money may give individuals a greater perceived right to control the money they earn (‘my money’) in the workforce (Rake & Jayatilaka, 2002; Vogler, 2005), conflicting with the concept of an equal partnership (‘our money’). Further, household finance is often an uncomfortable topic for couples and families to discuss, leading to conflict in the most egalitarian of relationships (see Britt et al., 2017, among others). The so-called ‘money taboo’ prevents open discussion of personal finances, including household financial matters (Atwood, 2012; Sanders, 2015).

Complexity in the various layers of household money matters enables controlling partners to have financial control, often with severe consequences for victim-survivors and their children/dependents (Scott, 2020b). Seemingly benign methods of household money management can exacerbate unequal power dynamics, with institutional practices (including systemic biases) enabling abusive partners’ financial control. Examples of the mechanisms used by couples to manage their money and financial matters include (but are not limited to) individual and joint bank cheque, savings and loan accounts; joint debt outside banks (for instance, utilities, other financial services including third-tier lenders and finance companies, or ‘pay later’ schemes); investment ownership (property and other financial assets); family trusts; and primary residence ownership. In each instance, external organisations use their own and industry-standard policies and protocols to regulate and govern the use of these tools by an individual and/or couple.

The banking sector especially has a stake in the prevention of and response to financial abuse (Sharp-Jeffs, 2015), given their almost-total reach across global populations (World Bank, 2018) and intimate knowledge of a household’s financial matters. In this paper, I extend existing understandings of ‘systemic harm’ to motivate the bank’s role in directly addressing economic harm in the context of IPV, moving beyond economic stability and corporate social responsibility (CSR) work dominating banking, financial and corporate ethics.

### The Experience of Financial Abuse as Consumer Vulnerability

Hill and Sharma (2020, p. 551) define “consumer vulnerability as a state in which consumers are subject to harm because their access to and control over resources are restricted in ways that significantly inhibit their ability to function in the marketplace.” This definition is appropriate here for two primary reasons. First, it is general enough to apply to any industry and thus adaptable to banking, financial institutions, and/or services. Second, and arguably more importantly for our purposes, it echoes the definition of financial abuse, provided earlier, almost word for word. This latter reason also highlights an important distinguishing factor for victim-survivors of financial abuse and IPV more widely, from other life experiences and circumstances commonly understood as potential points of vulnerability. Victim-survivors are subject to both the intentional actions of their abuser *and* those unintentional actions of their financial institution. Both forces exacerbate a victim-survivor’s experience of vulnerability and directly impact “their ability to function in the [financial] marketplace”.

The definition above also provides instructive scope for any organisation seeking to build actionable policies and practices and provide practical guidance for their staff. Namely, despite the introductory section of this article stating that intersectional disadvantages compound financial abuse and IPV, disadvantage alone does not render a consumer vulnerable. “Disadvantaged groups are disadvantaged because they are unequal…in a specified context” (Hill & Sharma, 2020, p. 554); however, disadvantage alone does not automatically ensure that an individual is vulnerable to harm from an organisation (here, a bank). There is a difference between disadvantage (i.e., one’s characteristics) and vulnerability: the circumstances experienced by an individual that adversely impact their ability to control and/or have access to resources and/or a marketplace. That is, circumstances opening someone to harm are what render them vulnerable, not any particular disadvantage they face. Vulnerability is thus predicated on the interaction an individual has with another party or organisation. By definition, all those experiencing circumstances rendering them vulnerable are open to harm from those outside parties (banks) that have the power to restrict their autonomy and agency in a (financial) market. However, harm arising from an individual’s vulnerability is not binary nor guaranteed and exists on a spectrum—a note important to remember when prioritising financial abuse over other areas of potential vulnerability banking customers face.

The adverse consequences for institutions behaving poorly with their so-called ‘vulnerable’ consumers have recently been highlighted, gaining the notice of professional bodies, regulators, and financial institutions alike. For instance, Australia’s (Hayne) Royal Commission into Misconduct in the Banking, Superannuation and Financial Services Industry has led to significant legislative and regulatory change across the sector. In the N.Z. context, third-tier lenders (including consumer finance companies and ‘truck shops’) have come under scrutiny for predatory lending practices. They are now subject to stricter lending rules under the amended Credit Contracts and Consumer Finance Act (Consumer Protection, n.d.). Protecting individuals facing not disadvantage but life circumstances leaving them vulnerable has been highlighted as essential to ensure an equitable and inclusive society. Such ethical discussions are not new, with marketing practices that may be considered exploitative (‘target marketing’) and their ethical implications considered for decades (see, e.g., Craig Smith & Cooper-Martin, 1997). The stakes of getting it wrong are high, nor is it only a problem for the finance sector, as demonstrated by the recent AUD 50 million fine for telecommunications company Telstra, for predatory sale practices and “unconscionable treatment of Indigenous phone…customers” (Bainbridge & Thorne, 2021).

Where does this leave victim-survivors of financial abuse? Violence is undoubtedly traumatic to live with and experience. However, research finds that women do not necessarily feel vulnerable until they need to seek help from outside organisations (Wilson et al., 2019). For victim-survivors of financial abuse, their vulnerability in relation to their financial institution may not be felt until they are required to interact with their bank or a budgeting or debt service. For our purposes here, it is helpful to draw a line between the abuser’s actions creating the circumstances that make the victim-survivor vulnerable to harm and the harm inadvertently caused by their subsequent interaction with the financial institution/bank. Distinguishing between the two forces eroding a victim-survivor’s agency and inclusion in the financial marketplace is key to outline the bank’s role and response, ensuring further harm is avoided. However, when it comes to operationalising the ‘inclusion’ of customers experiencing circumstances that render them vulnerable, the implementation of novel codes of practice is more complex.

## The Case(s) for an Informed Banking Response to Financial Abuse

### Aotearoa New Zealand: Background and Context

Aotearoa New Zealand (N.Z.) provides a unique setting for exploring the role of retail banks in responding to financial abuse in the wider context of IPV. Intimate partner violence (IPV) rates are among the highest globally (see, e.g., Rutherford, 2016) and the highest in the developed world. N.Z. data places lifetime prevalence at 1 in 3 N.Z. women experiencing physical and/or sexual violence (a caveat to N.Z.’s statistics may be a high reporting rate relative to other nations, or higher rates of IPV, or both). When one includes psychological abuse, this statistic increases to 1 in 2 women (New Zealand Family Violence Clearinghouse, 2017)—under N.Z. legislation, economic and financial abuse is categorised as psychological abuse. No economic and financial abuse population-based prevalence data exists for N.Z. at the time of writing. However, recent research found IPV-related financial abuse doubled in prevalence between 2003 and 2019 from 4.5 to 8.9% (Fanslow et al., 2021). Despite the Anglo-centric focus of the examples provided here, it is worth highlighting that Māori women are over-represented in IPV statistics (more than 1 in 2 Māori women have experienced physical and/or sexual violence over their lifetime, compared to 1 in 3 for non-Māori: see Fanslow et al., 2010). In terms of the pandemic, normality has mostly resumed although select industries (predominantly tourism, hospitality, universities) bear most of the economic burden. As do Indigenous peoples and minorities worldwide, Māori and Pasifika communities face additional structural inequities, including employment and health inequality, impacting their resilience to economic shocks (Kukutai et al., 2020).

When it comes to banking, various banks approach domestic and family violence differently. For example, some may have a dedicated in-house team to provide a ‘one stop shop’ for victim-survivors, while others provide information on their website. However, the N.Z. banking sector has no governing code of practice or guidelines to provide a framework for responding to economic and financial abuse or IPV more generally, unlike Australia (Australian Bankers’ Association, 2016) or the U.K. (U.K. Finance, 2018). Finally, N.Z. households are more likely to have an interest in a family trust entity (or entities) relative to those in other Anglo-Western nations (Law Commission, 2013), adding a layer of complexity to money management and property settlement when an intimate partnership ends.

### A Note on Methodology

To illustrate my conceptual argument, I offer two cases of banking experiences: one negative (Anna) and one positive (Eloise). Both stories highlight the tangible impact banks can have on the experience of women facing violence, their financial security both during their ‘relationship’ and post-separation, and the challenges financial institutions face in providing support for victim-survivors. In writing this article, I have opted to outline where improvements are crucial to avoid furthering economic harm of vulnerable victim-survivors *and* juxtapose harm with good. The scope of the discussion is retail banking, however, the understandings of vulnerability in the context of financial abuse are relevant beyond the financial services sector. Names have been changed, and some details omitted to protect participant anonymity.

Anna and Eloise’s stories were each selected from twenty-three interviews collected in two larger qualitative studies, undertaken in 2018 and 2019/2020 respectively, to examine what *post-separation* financial abuse looks like from women’s experience (in N.Z.). Both larger projects were designed and developed in alignment with a Constructivist Grounded Theory (Charmaz, 2014) methodological approach, deemed suitable for the wider research agenda the studies sit within and the critical social justice perspective taken by the researchers. Constructivist grounded theory “provides tools enabling researchers to go deep into studied life and see it from varied vantage points” (Charmaz 2020, p. 167). The flexibility inherent in the approach allows continuous analysis during data collection, the culmination of which may be new theory and understandings on a topic whilst the research is underway, described by Charmaz (2020, p. 167) as prompting “new ideas, revised directions, and can lead us to retrace our steps”. The approach is therefore iterative, as questioning assumptions may lead to further data collection to verify findings and resultant theory. For those interested or unfamiliar with the approach, Väyrynen and Laari-Salmela (2018) provide a useful overview of grounded theory in business ethics research.

Both the 2018 and 2019/2020 studies focused on women’s experiences, and I use gender-specific language here, as IPV is predominantly gender-based male violence against their female (hetero)romantic partner (World Health Organisation, 2010). However, violence is also experienced in non-heterosexual relationships (see e.g., Stark & Hester, 2019), and men can be victims of IPV in heterosexual relationships. I acknowledge these experiences as important; however, they were not the subject of the studies described here. The purpose of the current article is not to present comprehensive findings of the larger studies but instead to briefly describe the contrasting banking experiences of two women, both of whom recounted complex stories of IPV. Further details of methods are available in Vogels and Scott (2020). Dr Christina Vogels collaborated with me on the 2018 project, with interviews and fieldwork completed in partnership. Her contribution is gratefully acknowledged.

This section will proceed as follows. Qualitative data collection and participant recruitment for the two larger studies (from which the two cases are drawn) are described to provide context for Anna and Eloise’s interviews, followed by my self-reflexive positioning (integral to any qualitative work). Preliminary analysis follows, providing a foundation for selecting Anna and Eloise’s stories.

#### Data Collection

Women were invited to participate in the 2018 and 2019/2020 studies through two advocacy organisations, which acted as crucial conduits between participant women and the researchers. For the 2019/2020 project, some women contacted the author directly following media interest in the earlier study. For eligibility, we required women to be permanently separated from their former partners. The ‘snow-ball’ purposive sampling approach ensured recruitment could be carefully managed in terms of the number of potential participants so that no eligible woman was refused a place in the study due to time or funding resource constraints. The inclusive nature of the approach was ethically important to the researchers, given traditional silencing of women’s stories of violence. While the recruitment strategy sought to avoid further silencing participant women and their voices, I acknowledge we primarily spoke to those women selected by the advocacy organisations.

Twenty-three women were interviewed: fifteen over four months in 2018 and a further eight in late 2019 and early 2020. Qualitative methods are appropriate to capture the complexities of lived experiences of IPV and, specifically, financial abuse. Interviews were semi-structured and lasted between 90 and 120 minutes, with each woman provided the opportunity to recall her experience of violence both during and after the ‘relationship’ ended. Interviews were audio-recorded and later transcribed by one of three research assistants (except one transcribed by me), then collated with any additional documentation provided by participants. Transcripts were read for accuracy and each interview summarised in preparation for further analysis. Anna and Eloise, the cases presented in this article, were two of the twenty-three participant women interviewed.

#### Researcher Reflexivity: A Qualitative Requirement

Each successive interview challenged my prior understandings of the role banking institutions have in the private lives of individuals and couples. Charmaz (2020, p. 165) asserts constructivist grounded theory’s “emphasis on reflexivity…prompts us not only to examine who we are in relation to the research but also to remain reflexive about how we use grounded theory strategies” (p. 165). I therefore offer my self-reflexive statement here, as prelude to the preliminary analysis section that follows.

Trained in finance, both academically and professionally, I am fascinated with how money and financial resources influence behaviour and shape individuals’ lives. Societal-level factors, including the money taboo, gender pay and investment gaps, and the ownership we may place as individuals on ‘our money’, prevent open and constructive money conversations and can contradict the equality we look for in our intimate relationships. When paired with the emotional complexity of intimate partnerships, it seems logical that unequal power dynamics are exacerbated by inequity in financial resources. By extension, in the context of IPV, money and financial resources can be used as weapons of entrapment against an intimate partner (not exclusively, but predominantly, male violence against women).

A white New Zealander (Pākehā), raised in Australia, I am a university-educated working mother of young children, who assumed the role of ‘stay at home’ mother initially and now, am primary breadwinner while my husband is the primary carer of our children. Overlaid by studying and working in a male-dominated profession, each of these roles influenced my collection and interpretation of all stories collected during the study. My analytical and emotional response to the research project has evolved and matured over time, as my professional background has strengthened to include wider understandings of how money is used to exert control over another (see Vogels & Scott, 2020 for an in-depth exploration of the emotionality of researching financial abuse). For instance, it has taken some years to fit comfortably within the identity of ‘feminist’ and at time of writing, ‘pragmatic feminist’ is closer to the mark (Alfonso & Trigilio, 1997). Exploring fluid researcher identities is left for a future article and mentioned here solely for the purposes of full and candid self-reflexive positioning.

#### Preliminary Analysis: Banking Relevance to Experiences of Financial Abuse

The semi-structured interviews resulted in in-depth narratives recounting women’s complex lives and experiences of IPV. The focus of the 2018 and 2019/2020 studies described in the ‘Data collection’ section above was financial abuse. Specifically, the ongoing impact it, the abuser, and institutions (including banks and the family court) have on women’s lives as they rebuild them after their ‘relationship’ with the perpetrator ends. Given our focus on money management and finance, participant women were asked about their banking arrangements during and after their relationship. Of the twenty-three women we spoke with, approximately one quarter (*n* = 5) redirected the conversation at this point, or stated the bank was not a factor and/or indicated they had not disclosed nor thought to disclose their situation to their bank. Such redirection was not unusual over the course of the twenty-three interviews, as women sought to tell the researchers *their* story, *their* way and interwove their narratives of financial harm with other forms of violence. Somewhat jarringly (for me, at the outset of the 2018 project) the financial lives participant women lead could not be easily disentangled from the wider environment of violence they face, either during their ‘relationship’ or afterward (Vogels & Scott, 2020). This complexity of context and experience underscores the importance of the conceptual argument made in this article, positioning the bank as (systemically) important to a consumer rendered vulnerable by their experience of (financial) violence.

Eighteen women discussed their experience with banks as either directly or indirectly relevant to their experience of violence, often briefly (answering the question asked, then redirecting the conversation as above) with a relative minority responding in detail/at length. Anna and Eloise’s narratives were selected from this smaller group of eighteen.

#### Case Selection: Anna and Eloise

Participant women we spoke to overwhelmingly (*n* = 17) related negative banking experiences during our talk. Of these, three alluded to (either directly or indirectly) the arguably nefarious intent of individual actors at the bank. While perhaps indicative of poor organisational practices and processes, this article remains focussed on industry and institutional level systems and policies, seeking not to focus on any one bank or financial institution or actor within that organisation. These three stories were thus excluded from case selection in this article. Of the fourteen remaining ‘negative’ stories, Anna’s interview explicitly documented the *unintentional* harms financial and banking institutions cause via their pre-existing policies, practices, and systems. She highlights many of the elements contained within the interviews of other participant women, bringing together the tentative codes and preliminary understandings built over previous interviews and illustrates the cumulative impact of successive institutional actions. Anna’s articulate recount draws direct links between the abuse, bank actions and how the real consequences played out, clearly demonstrating the urgent need for action by organisations to respond to financial abuse and IPV, more generally.

Some participant women named a comparatively better experience with one bank over another (usually much more negative) banking experience. However, of the eighteen who discussed banks as relevant to their experience of abuse, only *one* woman was emphatically positive when discussing her bank. In a short passage of our interview, Eloise’s narrative illustrated the ‘tone’ any institution can take when seeking to avoid doing further harm to the victim-survivor. More than any other participant, Eloise outlined how the bank helped and the corresponding impact on her experience of violence. Her story lays foundational groundwork toward building supportive understandings of victim-survivors as consumers facing vulnerability. For those women who compared one banking experience to another, usually contrasting positive with negative during their interview, the importance of an empathetic tone was apparent—regardless of whether the victim-survivor had disclosed their circumstances to the bank. Eloise’s banking experience, while unique in terms of the other interviews, clearly points toward the direction we might take in building better banking responses—and provides wider guidance for all organisations looking to define their role in combating IPV.

To illustrate the complex lived reality of both women, Anna and Eloise’s experiences of violence and their subsequent banking interactions, I present each as a vignette: a short summary of their experience. Doing so allows focus on the elements of their narratives most relevant to the topic of this article (financial abuse as IPV and the relevance of financial institutions to that experience) whilst retaining the wider context of their lives, as relayed to the researchers. While contrasting markedly, when Anna and Eloise’s stories are taken together, an understanding of why and how (financial) institutions must respond to financial abuse in the context of IPV can be built.

### Anna: An Example of a Poor Banking Response

Anna, a mother of four, spoke of two relationships during our talk. The first, her 21-year relationship with her former partner, is the focus of this case. I will use minimal commentary, preferring to use Anna’s words where possible, although the potential points of bank intervention are emphasised.

Anna had been with her partner for 14 years before they had children. She says the relationship had always had “moments” of violence, but that “she didn’t really know because a lot of the times you don’t know it’s a controlling, abusive relationship”. Their finances were completely joint, including bank accounts and two rental properties—everything in both names. They were never married. Anna pointed to a change when she had children, as “when it started to get quite dark”. She stopped working and says “there’s a sense of entitlement” to him becoming the sole breadwinner. Money became tight, and she did not have enough.

As her pre-children career in hospitality was no longer practical, Anna opted to find other sources of income, including study. She said, “Okay, I can study, but that is when he started getting violent…I went to sit my first exam management 101. I think it was five essays, and … he broke my hand.” Then, he opened a separate bank account and got wages put into that—Anna does not know when he did this; she guesses when she started having children. Anna had been seeing a counsellor and recognised the abusive nature of her relationship—she says, “You plan, to get out of abuse you have to plan.” For Anna, that involved staying in the relationship until her broken ribs had healed, as she knew she would struggle with four young kids at home (the youngest were twins). For brevity, by 2006, Anna had a protection order, although she distrusted organisations she’d had contact with, including the police, lawyers and other government institutions.

“So, I start to feel really scared. Scared of her [the lawyer], scared of the police, scared of him, scared for my kids.”—Anna.

For example, the lawyer suggested she take the joint credit card to pay the legal fee incurred in obtaining the protection order, NZD3000, and Anna refused: “I can see that being used against me, for clearing out the credit cards like that.” On another occasion, the police asked what she had done to make him so angry that he would breach the order. Her abuser went on to breach the protection order “probably every couple of months for seven years.”

In terms of banking, Anna was on the Domestic Purposes Benefit for those seven years and “never missed a mortgage repayment”. Her former partner was not contributing to the mortgage or maintenance on the family home, nor paying child support. At one point, when she estimated they had about 50% equity in the family home, she requested an NZD10,000 loan to replace leaking windows. Her abuser refused to sign the documentation from the bank to extend the loan.

Later, she applied for a mortgage holiday as she wanted to work now her twins were at school. The mortgage holiday would enable her to repay her credit card debt of approximately NZD3000, and she could start work without the debt burden. Anna explained her situation to the bank employee; he provided assent. She filled out the paperwork and, 2 weeks later, was declined. Upon querying why Anna was informed she was on welfare. When Anna challenged their reasoning, given she had never missed a repayment, the bank advised she required her former partner’s signature. Anna explained she would try but that she did not think he would sign. He did not. In her words, “I had tried on three occasions to separate from him financially”.

The bank then advised Anna to “stop paying the mortgage, and we’ll go for him, that might make him start the proceedings.” When I asked Anna how she felt about that advice, she told me she was sceptical at first, but in the end “trusted the bank manager” and stopped paying. When her former partner arrived home to N.Z. from a stint working overseas, his bank accounts were frozen. Anna says the bank didn’t tell her they would freeze his accounts, and had she known, she would never have asked for the mortgage holiday nor stopped paying. I asked why. In Anna’s words, “He would do something…I mean this guy had the potential to kill, he was that type of person.” Out of fear, Anna then went home to pay the mortgage up to date. She had no money in her account to do this (Work and Income New Zealand had stopped paying her benefit on a tip-off, a mistake they rectified some months later).

The bank’s advice had consequences. Not paying her mortgage allowed Anna’s former partner to tell the court she was forcing a mortgagee sale on the family home and was therefore playing games. The judge ordered the protection order lifted and the children to remain with their father. The house was ordered to be sold via an agent. Despite claiming to the court he had no money and therefore needed the house sold, her former partner bid on the house at auction. Anna eventually settled in the Family Court for much less than her lawyer thought she should have, to have it over and done.

Anna says, “I needed counselling…none of that was available. I had no money. I needed to understand the dynamics of abuse. I was hugely vulnerable.”

Unfortunately, Anna then met a man online, and the vignette related here is not the end of her story.

### Eloise: An Example of a Better Banking Response

Eloise, a mother of two adopted daughters, was mid legal battle at the time of our talk—her story, as written here, illustrates the severity of harm faced by victim-survivors and its ongoing and long-term nature. She met her former partner, Edward, overseas and married him one year later, returning to N.Z. while he stayed in his home country. When he later joined her in N.Z., Edward was “really upset” she did not own a house. Eloise is unsure why he thought she did, as she had not indicated she owned property.

To appease Edward, Eloise bought a house, originally under the impression it would be under both of their names. Edward refused, insisting they have a contracting out agreement (pre-nuptial agreement), as was custom in his culture. Doing so meant his assets overseas were partitioned from Eloise’s in N.Z. (she purchased a second property in addition to her home). Before the agreement was signed, Edward decided he would return to his home country to visit his mother. When Eloise advised she did not have the money to send him, he “smashed [her] head into a wall”. Unable to work or complete her study, Eloise separated from Edward and moved into her property, getting a flatmate to help cover the mortgage as she could no longer work full-time. Following their first separation, the marriage became somewhat on-and-off, with Eloise funding Edward’s lifestyle, trips to his home country and sending money to his mother and friends. However, Edward became increasingly violent. Eloise explained various situations where Edward would become enraged, including if she walked past him as he prayed or prepared food he did not like, and physically assault her.

“I remember when he had his hands around my neck, I stopped struggling. And it was a conscious decision, you know. And immediately fear left me, the panic or the fight for life just left me. That survival instinct just left. … And he stopped squeezing. When I gave up, I think he must have thought that he’d already done the job. … I think in that moment, if I hadn’t believed, all that rubbish [I deserve to die. And everything he says is right] and not relaxed, I’d be dead. And still, I kept throwing money at him so he could see his mummy.”—Eloise

Eloise detailed several specific events in addition to her quote above, including one where she asked him to look at her camera as it was not working, and he smashed it on the floor in front of onlookers. She was so embarrassed she separated from him immediately, although he remained in the house and paid her a nominal amount in rent. Despite her re-applying for divorce, he wanted to reconcile—Edward’s visa needed renewal, and he asked Eloise to halt divorce proceedings. She did.

Regarding their financial arrangements, Eloise says,

“He had his own account. I had my own accounts. So, he had his accounts, which his money went into. He spent his money. And I had my accounts, but I could only spend my money the way that he told me to spend my money. So, I couldn’t go out and buy new clothes or take the girls to a coffee shop or go out for a meal unless, well I couldn’t. He just wouldn’t give me permission for that. So, his money was his. My money was his. Even though we had separate accounts.”

Explaining her experience of entrapment, she elaborated,

“You might have account in your name. That doesn’t mean, you have control over your finances. Doesn’t mean you are not going to get beaten within an inch of your life if you dare buy a cup of coffee or a fluffy for the children. You know, ten dollars compared to the tens of thousands of dollars that he takes out.”

Eloise remembers the time Edward was overseas fondly, despite him using her money to travel, explaining during these periods she felt she was independent, able to use the phone freely and go for walks. However, when he returned, it would be 3 weeks of hell as Edward re-adjusted to N.Z. life. Eventually, following the adoptions of their two daughters, Edward agreed to separate from Eloise. Less than a year later, he approached her for NZD7000 to go overseas. Eloise gave him the money. They had commenced separation proceedings, and Edward placed a caveat on Eloise’s house, claiming it was relationship property and that due to his poor English, he had not understood the contracting out agreement (pre-nuptial agreement) he had signed. The caveat meant that she could not sell the property, and despite Edward promising to remove the caveat several times over the 2 years that followed, he has not.

Three years after their final separation, Edward almost succeeded in killing Eloise. Police were called; however, Eloise was advised not to go to court as “they’re going to crucify you” because “you could say, anyone’s done this”. Instead, they suggested she seek the help of support services (their commanding officer later apologised for the officers’ inappropriate response to the assault). While Eloise said attending a family violence service and meeting other women facing similar circumstances has helped immensely, she is angered any of them have experienced what they have.

Post-separation, Edward refused to pay child support. Despite the Family Court acknowledging the abuse Eloise and their daughters have suffered, at the time of her interview, Eloise was concerned his claim of fifty per cent of the value of the property plus legal fees would be awarded. To fund her own legal expenses, she took a mortgage out on one of her two properties, which ended up going to a mortgagee sale.

Over the separation proceedings, Eloise credits her bank as getting her through: “I’ve never dealt with such a terrible situation with such lovely people.” She appreciated the credit controller kept her updated on what she could expect, such as a bailiff arriving, and reassured her throughout the process. However, Edward’s lawyer halted the proceeds of the mortgagee sale being released and eventually, her bank settled with her former husband to release the funds. The money from the sale (less the settlement) was used to purchase a new property, and her loan was restructured. The bank allowed Eloise to pay interest only on the mortgage. When the interest-only payments come to an end, Eloise will need to pay the mortgage in full (which she will be unable to do), and the house will go to a mortgagee sale.

At the time of our conversation, Eloise advised Edward had had her accounts frozen—she believes with the intent to ensure she cannot provide for herself or the girls. She lived off the generosity of friends and was working 80-hour weeks. She had formulated a plan for the care of the girls once she lost her home, knowing her credit rating was too poor to rent a property and was facing homelessness herself.

“It doesn’t mean that you’ve got your own name on your bank account, that you have financial freedom. Or you have control over your own life or what you spend. And that has been the hardest lesson.”—Eloise

Eloise has stayed in sporadic touch with me since her interview. She and Edward are still in the Family Court, and proceedings to financially separate from him remain ongoing.

## In Brief: Unpacking Trauma

To unpack what is going on for Anna, Eloise, and other victim-survivors of financial abuse, I will first revisit the concepts of entrapment and vulnerability expounded on above. Second, I relate the experience of financial abuse as consumer vulnerability to our bank and financial institution setting (a line of argument one could also apply to other consumer-facing businesses, such as telecommunications or utilities). Finally, referring to Herzog’s (2019) ‘narrow’ and ‘broad’ view of systemic harms, we can operationalise the consumer vulnerability of an IPV victim-survivor into two categories: the narrow, or ‘do not make it worse’, and the broad, or ‘attempt to make it better’. The former is reactive, whereas the latter is a proactive stance. Considering the bank as a systemically powerful institution both at a macro (societal) and micro (household) level is helpful as we conceptualise the financial institution’s response to individual disclosures of violence by existing customers and any preventative role in society’s response to IPV they may play.

Economic abuse (and financial abuse or financial violence/control, more specifically) is the use of financial resources, including employment or education, to control an intimate partner. Coercive and controlling relationships rarely appear obvious to outsiders, and victim-survivors may not immediately realise they are being abused. Victim-survivors, by definition, may not have the level of independence and agency to make decisions benefitting themselves and their families—they may not act in ways outsiders think they can or should. Using a lens of consumer vulnerability, an organisation may recognise a consumer’s circumstances render them vulnerable to harm. However, the victim-survivor may not perceive they are vulnerable until the interaction with the organisation leads them to feel this way or know their vulnerability.

In Anna’s case, she simply wanted to be kept safe from her abuser. The systems in place were inappropriate for doing so. In Eloise’s case, her bankers responded to her situation with empathy. They made her feel as though they were doing everything they could, within the parameters of their organisation, to support her. Through these two examples, a clearer picture of both what it means to be vulnerable and how a financial institution can respond emerges.

### A ‘Narrow’ Banking View of Systemic Harm

There have been instances of privacy and system failure in a banking setting, including disclosing a victim-survivor’s new address to her abuser (Austin, 2019). Such examples are often of operational failure (a note or update not read) and result in internal reviews of procedure to ensure the error is not repeated in the future. Arguably more insidious, although no less severe, is outside advice that causes harm. Two examples are present in Anna’s story. The first is a practice that continues today: the bank requiring victims to re-engage with perpetrators to fulfil policy and procedural requirements—for example, requiring both signatures for all changes to a loan or account. For Anna, the bank required her to re-engage with her abuser to make autonomous financial decisions to benefit herself and her family. The second example is when her abuser blocked her attempts to financially separate from him; the advice she received was inappropriate given the legitimate concerns she had for her safety.

Unlike in Eloise’s case, where each step of the process was outlined to her, for Anna, the implications of accepting a banker’s actions were not spelt out. Victim-survivors are the experts when it comes to keeping themselves safe. When weighing the advice of professionals or outsiders, it is crucial for a victim-survivor to know exactly what will happen should they assent to a specific action or procedure, so they can effectively assess the risk the action may pose to their safety. While being financially capable may help, it cannot prevent financial abuse alone. Financial literacy programs aimed at IPV victim-survivors see significant increases in confidence (self-efficacy) rather than money management skills (Sanders et al., 2007). Anna showed she was financially capable again and again, even in the face of poor advice from professionals, including her bank. On the advice to stop repaying her mortgage, Anna told me she has since heard she is not unique; it is not uncommon advice.

**Fig. 1:**
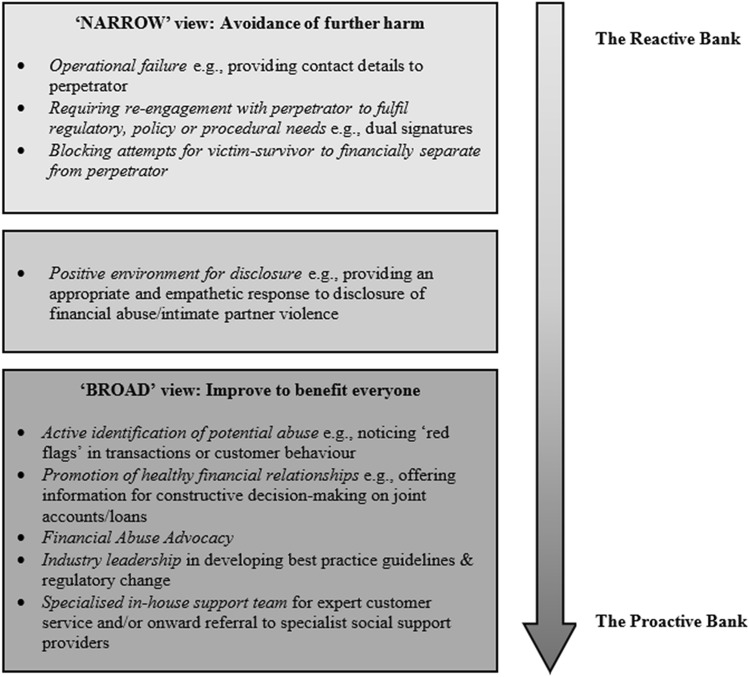
Systemic harms approach to financial abuse in the banking context

The ‘narrow’ view of potential harms presented here are reactive from a financial institution’s standpoint (Fig. 1). The goal in designing policy and procedure is to ensure an existing customer’s circumstances are not made worse through the bank’s actions. That is, the consumer is not rendered vulnerable by the system with which they are interacting. Referring to Hill and Sharma’s (2020) definition of consumer vulnerability, an informed set of policies and practices prevents harm for the victim-survivor of financial abuse arising through their interaction with the bank. That is, their ability to function in the financial marketplace is not controlled or restricted by the financial institutions and their systems. Returning to the problem inherent in re-engaging with perpetrators, changing bank policy only to require dual signatures if the outcome is not beneficial to the customer is possible, for example, the opportunity of a lower interest rate on a loan product. In this case, the bank could simply lower the interest rate and inform the customer of their action. For cases where dual signatures are required for regulatory reasons, measures can be put in place to ensure bankers have a full set of information available to help their customer keep themselves safe. However, a narrow view of systemic harms, that is, the avoidance of further harm, is unlikely to be enough in this case. Instead, a proactive or ‘broad’ approach, and attempt to ‘make it better for all’ may be required.

### A ‘Broad’ Banking View of Systemic Harm

For customer-facing staff to appropriately serve their customers, awareness of what coercive control may look like in a banking setting is required. Research shows positive social reactions to IPV disclosure (e.g., validation and provision of support/help) are important to victim-survivor’s mental health and general wellbeing (Woerner et al., 2019). Therefore, financial professionals need education and training to address direct disclosures of financial abuse or IPV and identify potential red flags. The latter is arguably where this approach becomes proactive, in line with Herzog’s (2019) concept of broad systemic harm. Rather than simply providing a safe environment for victim-survivors to disclose abuse, dealing with the customer’s circumstances and their potential vulnerability sensitively (‘do not make it worse’), there is scope for financial institutions to help consumers identify that they may be the victim of financially abusive behaviours and offer support.

A broader view of banker responsibility also allows general bank messaging to actively promote healthy financial relationships between couples at key junctures, such as taking out joint debt or opening joint accounts. Banks may already be involved in community outreach, such as financial capability education in schools or sponsorship of community services. Therefore, it is not entirely beyond their remit to take a more prominent advocacy role. Industry leadership in the design of best practice guidelines for regulators to rollout industry-wide also falls under this ‘attempt to make it better’ response to financial abuse as a social problem requiring collective action, such as the Five Point Plan developed by The Co-Operative Bank in partnership with Refuge in the U.K. (The Co-Operative Bank, 2020). Outside the banking context, immediate pandemic-induced need for government, industry, and business-led crisis responses have also led to innovative ways for IPV victim-survivors to report violence. Examples include the use of code words in essential retailers (Home Office, 2021), providing free internet/wifi in essential stores to improve access to support services and emergency services promoting ‘silent solutions’ for those seeking their help but unable to safely speak (Ministry of Justice, 2021). ‘Safe exit’ buttons on a website describing financial abuse may further protect victim-survivors from their abuser monitoring their browsing history, a technique any organisation can implement.

Finally, a proactive bank or financial institution may form a specialised in-house team for customer-facing staff to refer customers to in the event of disclosure of violence or suspected violence (Fig. 1). In Aotearoa N.Z., BNZ’s Customer Assist team contains a specialist economic harm response unit (Edmunds, 2020). In the U.K., examples include RBS NatWest and Lloyd’s Banking Group (Surviving Economic Abuse, 2019). This team may have relationships beyond the organisation with family violence support services (highlighted as crucial by Surviving Economic Abuse, 2020) and offer specialised banking assistance to existing customers and potential customers. Victim-survivors ending a relationship with their abuser may desire to establish a new banking relationship with another institution for a clean start, separate from their former spouse. In these cases, a proactive and ‘broad’ stance may be to have guidelines regarding the necessary documentation required to open a new account, sensitive to the fact a victim-survivor may not have their identification documents available to them. For existing customers, debt liabilities incurred due to violence or coercion may be partially or fully waived. Ultimately, operationalising any comprehensive consumer vulnerability practice within an organisation requires informed policies and procedures that adhere to regulatory requirements while being open enough to allow bespoke solutions to mitigate vulnerability and systemic harm.

### The Bank is Not the Entire Solution: Signposting and Referrals

To appropriately address financial abuse, banking staff require clear tools *and* certainty over which aspects of a customer’s situation they can and should deal with. These are the actions they need to take to prevent consumer vulnerability and harm arising due to the banking transaction at hand. However, not all of a customer’s circumstances fall under the banker’s responsibility or the care they ought to afford their customers. In the context of IPV and financial abuse, the bank does not have the expertise to support the customer beyond their immediate interactions with the financial system. Therefore, the bank requires clear referral policies allowing staff to refer their customer onto specialised services, such as family violence agencies, to receive holistic support. Further, a bank may provide a grant or financial resources to ensure customers can access support (such as that provided by National Australia Bank, nd).

## Ongoing Challenges

Intimate Partner Violence (IPV) is an undeniably complex social problem, requiring a coordinated and collective response from various fields, including legal and justice, health, government agencies *and* financial institutions—indeed, any consumer-facing organisation dealing in economic resources (e.g., utilities). Economic and financial abuse is experienced by almost all victim-survivors of other forms of coercive control and violence, as one of a range of control tactics used by perpetrators of IPV (Kutin et al., 2017 find economic abuse is “significantly associated with other forms of IPV” [p. 269], citing a range of international IPV prevalence studies). However, coercive control can be experienced without the physical violence society still tends to associate with domestic abuse. While our understanding of what constitutes family violence or domestic abuse is widening, economic and financial abuse sits within a grey area of poor awareness and is compounded by the money taboo and societal, gender and cultural norms. Here, I touch on a range of ongoing challenges for banks and financial institutions, including their joint customer relationship to both victim-survivor and perpetrator, and myths and misconceptions of staff.

### Joint Customer Responsibilities

Perhaps the biggest hurdle for financial institutions in unintentionally causing further harm to a victim-survivor of financial abuse is that the perpetrator is likely also to be a customer. This joint relationship means several conflicts may arise for banks and financial institutions around regulatory constraints, including privacy and transparency. Transparency in all relationships is key, as demonstrated in Anna and Eloise’s cases above, where their respective bankers offered varying levels of transparency around their actions, systems, and processes. For victim-survivors of abuse, transparency of their financial obligations and money arrangements within their intimate ‘relationship’ often does not exist. However, they are held liable for financial decisions made without their consent or under coercion. For bankers, awareness of the dynamics at play when presented with a case of financial abuse is critical to forming a response that satisfies best practices and reduces the potential for further harm. This hurdle is not insurmountable; however, it requires careful thought and informed policy to ensure procedures are clear for customer-facing staff.

When banks are acting on behalf of a victim-survivor, privacy and information-sharing limitations restrict the protective actions the bank can take to limit perpetrator abuse, such as transaction abuse (Brook, 2020; Newton, 2021). When the perpetrator of economic harm is their customer, the bank cannot contact the victim-survivor’s financial institution and disclose on their behalf, having to limit their action to a warning or, in severe cases, debanking the individual. Addressing these difficulties transcends the institution themselves, and their industry, with any shift in policy and procedure requiring regulatory (and in some cases, legislative) change.

### Myths and Misconceptions

Bankers are not immune to having the same implicit and explicit biases present in the general population, and misconceptions of what a victim-survivor of IPV looks like or the forms violence can take remain an issue globally (see, e.g., Karlsson et al., 2020). In general, banking and financial institutions remain male-dominated (World Economic Forum, 2017) and gendered in their service provision, despite moves toward gender equity (Forseth, 2005). For instance, victim-survivors may not fit the stereotypical ‘victim’ mould. A victim-survivor may not know they are subject to abusive behaviours and financial control. Financial abuse does not discriminate across demography, including gender and sexual orientation. For men, being the victim of IPV and financial abuse may be even more fraught (Dixon et al., 2020; Lysova et al., 2020). A person experiencing abuse may be from a wealthy household; they may be facing poverty. Household income or individual earning power is not always indicative of access to financial resources. A customer who is educated, put together, strong and smiling may be hiding a reality far from what they are letting the outside world see. Anna asserts she didn’t present as a victim. In her words, “There’s nothing victim about it. I’m a mother of four children. I had had enough of being beaten…I am just taking back my power. I had a protection order now; I didn’t need to be afraid of him…”.

The importance of awareness, education and training cannot be over-emphasised, as individual bankers and those in the finance industry are likely to have the same misconceptions of IPV as broader society. Specific training on what financial abuse looks like, how it factors into a wider domestic environment of coercive control, and IPV more generally, can help staff identify and respond to explicit disclosures of abuse, and trigger statements that are potential red flags requiring further information. For example, a customer’s throwaway comment about not having access to an account when they should, seeming surprised when presented with an account or debt, or not knowing one’s own private details like a PIN. It is these red flags that customer-facing staff are likely to hear and see, and having clear guidance as to what to do next allows them to respond empathetically and work in the best interests of their customer.

Despite not necessarily having an explicit policy or guidelines governing their approach, bankers can and do support their customers outside the ‘usual’ provision of the banking service. Similarly, other financial services, including debt advice services, can help victim-survivors rebuild their lives. Empathy goes a long way, as is evident in Eloise’s story above. However, without trauma-informed guidelines to fall back on, bankers may be asked to make decisions beyond their experience and training. In this case, the consumer experience is likely to be ad hoc, inconsistent and detrimental to a wider agenda of financial inclusion and quality provision of services to those consumers who may be experiencing circumstances rendering them vulnerable to harm.

## Concluding Remarks

The very question of whether financial institutions have any place beyond the direct services they offer—their core businesses of financial transactions and lending—is an inherently ethical one. Financial institutions increasingly recognise they have an active role in addressing the economic and financial harm inflicted on victim-survivors of IPV, with examples in N.Z., the U.K. and Australia. By the time you read this, further momentum will have built as international information sharing between scholars, NGOs, and industry expands via formal and informal networks. However, the place for financial institutions in responding to economic harm in the IPV context remains under-explored, both in theory and practice. This article has sought to provide a preliminary step toward filling this gap.

Here, I present an ethical argument for banks and financial institutions, and consumer-facing organisations more generally, in taking a greater role in an area not traditionally thought relevant to their business: economic harm in the context of IPV. I build on work of business ethicists before me, namely *consumer vulnerability* at the micro-level while accepting a macro-level risk of *systemic harm*, to consider what an operational framework of new and improved policy might look like. Victim-survivors are rendered vulnerable by the circumstances they face—entrapped by their abuser. However, it is not until they have their financial autonomy and agency restricted or controlled in the financial marketplace by the banking systems they must interact with that the financial institution’s relevance in their vulnerability comes to the forefront. By framing victim-survivors as customers experiencing vulnerability, banks and financial institutions can begin revising their policies and practices to prevent the unintentional harm existing policies may cause. Such work is already underway for other consumer vulnerabilities but requires specialised knowledge, training, and understanding.

The strength shown by victims-survivors, illustrated by Anna and Eloise’s stories, emphasises that it is possible to provide support within the context of their complex stories without presuming to be the entire solution. It is appropriate to provide support to a victim-survivor in a way that is practical, with immediate impact right now, within the confines of existing policy and regulation. Simultaneously, the institution may lead internal and external conversations on best practices moving forward to enact lasting change to those same problematic policies and procedures. By considering more deeply what works in addition to what causes harm, future research may expand on the suggestions and rudimentary roadmap outlined in this article to further illuminate the issue for those working in the banking industry. This article is the beginning of what I hope will be a rigorous scholarship examining financial abuse and IPV more widely, beyond the obvious systems and institutions traditionally associated as having some role to play in addressing this evasive, invasive, and significant global social problem.
